# Improved Aerodynamics of a Hollow-Blade Axial Flow Fan by Controlling the Leakage Flow Rate by Air Injection at the Rotating Shroud

**DOI:** 10.3390/e23070877

**Published:** 2021-07-08

**Authors:** Michaël Pereira, Florent Ravelet, Kamel Azzouz, Tarik Azzam, Hamid Oualli, Smaïne Kouidri, Farid Bakir

**Affiliations:** 1 Arts et Métiers, Institute of Technology, CNAM, LIFSE, HESAM University, 75013 Paris, France; florent.ravelet@ensam.eu (F.R.); smaine.kouidri@ensam.eu (S.K.); farid.bakir@ensam.eu (F.B.); 2 Valeo Thermal System, Advanced Engineering, Thermal & Thermodynamics, 78322 Le Mesnil-Saint-Denis, France; kamel.azzouz@valeo.com; 3 Ecole Militaire Polytechnique (EMP), Laboratoire de Mécanique des Fluides, 16111 Algiers, Algeria; azzam.tarik@gmail.com (T.A.); houalli@gmail.com (H.O.)

**Keywords:** axial flow fan, CFD, leakage flow rate, air injection, hollow blade, rotating shroud

## Abstract

Axial flow fans are used in many fields in order to ensure the mass and heat transfer from air, chiefly in the heating, ventilation and air conditioning industry (HVAC). A more proper understanding of the airflow behavior through the systems is necessary to manage and optimize the fan operation. Computational fluid dynamics (CFD) represents a real tool providing the ability to access flow structures in areas that measuring equipment cannot reach. Reducing the leakage flow rate, inherent in operation, by synthetic-jet techniques improves performance. This paper presents the CFD results performed on a hollow blade fan developed by our team. The leakage flow is controlled by blowing air from 16 designated circular holes and arranged on the fan shroud. We discuss the results for two rotational speeds (1000 and 2000 rpm) and two injection rates (400 and 800 L/min). The numerical results consistent with the experimental show, for the low rotation speed and high injection ratio, significant gains in power (53%), torque (80%) and leakage flow rate (80%).

## 1. Introduction

The leakage flow rate developed in the operating set has shown a great influence on the turbomachines. This flow induced by the pressure difference either between the upper and lower surfaces of the blades, or between the upstream and downstream side of the device, is responsible for the energy losses and the generation of noise. It is in this context that the authors plan to analyze the mechanisms generated by such devices. Boudet et al. [1] have numerically and experimentally investigated the leakage flow rate for an axial fan. They showed that the major contribution of sound emission is induced by the tip vortex. For a turbine, with Delayed Detached Eddy Simulation (DDES) and the entropy analysis, Li et al. [2] investigated the loss mechanism of the tip leakage flow. They showed that the viscous dissipation loss is the dominant factor comparatively to the heat transfer loss. In addition, Fischer et al. [3] managed to characterize the structure of the tip vortex by using a modulable frequency Doppler Effect velocity meter with high temporary resolution. Pogorelov et al. [4] attempted to identify this structure for an axial fan. It was shown that the reduction in the dimension of the tip clearance leads to the reduction in both the size of the tip vortex and the amplitude of the noise. Throughout a LES investigation, You et al. [5] emphasized and confirmed that the effect of this dimensions on noise generation and vibration. In addition, in the compressor cascade, Zhang et al. [6] studied numerically the influence of the endwall suction under different tip clearance size. The obtained results showed that the tip leakage vortex is influenced by a particular endwall suction with performance improvement. Significant reduction in leakage loss was obtained with small clearance and improvement limitation was noticed for the case large clearance. Lee et al. [7] discussed the influence of the height of the squealer rims for a turbine. A reduction of around 11.6% was obtained for the total pressure drop coefficient.

To reduce the leakage flow rate, various geometric solutions have been proposed. Wallis et al. [8] have dealt with the extraction of the work during the passage of the leakage flow through small vanes bladelets or turning device placed on the shell in the presence of radial barriers fixed on the casing. They managed to reduce the tangential component of the leakage flow. However, they highlighted the unsteadiness and complex structural effects of the flow at the tip clearance. Corsini et al. [9] studied the shape of the endplats placed at the end of the blades in order to change the orientation and the formation of the tip vortex. Aktürk et al. [10] improved the performance and reduction of leakage flow rate of an axial fan by means of the study of the five platform extensions of the profile on the lower surface.

Pardowitz et al. [11] employed a rotating shroud for clearance vorticity suppression. Longhouse [12] was able to reduce noise and improve efficiency by using a rotating shroud attached to the blade tips. This device prevents the formation of the vortex tip. In addition, it is a solution envisaged in so far as we want to maintain a fairly large clearance between the fan and the housing. For electric motors, cooling is generated by a centrifugal rotor, Vad et al. [13] redesigned the reference rotor by adding a shroud. For the same cooling performance, it reduced fan noise and power consumption. In addition, some geometric solutions focus on optimizing labyrinthine joints. Schramm [14] presented an optimization method for the labyrinth seal in the aim of reducing the leakage flow.

This article is dedicated to the study of the leakage rate for an axial cooling fan of an automobile engine. We study flow control by injection. Several authors have sought to improve the performance of turbomachines by means of this technique. By injection upstream of the rotor of a transonic compressor, Weigl et al. [15] succeeded in stabilizing the rotary stall and surge with an expansion of the operating range. Even at the profile scale, Rhee et al. [16] increased the lift of an injection hydrofoil near the trailing edge.

Eberlinc et al. [17,18,19] managed to increase the coefficient of the total pressure by 6% of an axial fan composed of hollow vanes by injection near the level of the trailing edge at the end. The jet, which is caused from the hub by an internal flow line, allowed the control of the boundary layer by increasing the local velocity and reducing the effect of the adverse pressure gradient.

Neuhaus et al. [20] managed to reduce the leakage rate and improve performance with an injection rate of 0.8%. In addition, these improvements were noted for a pulsed injection synchronized with the rotation of the fan. They achieved with an injection rate of 1.5% to widen the operating range of the fan by 62% by pushing the stall point towards low flow rates. At the displaced point, the improvement in pressure was approximately 40%.

The study by Morris and Foss [21] was devoted to the automobile cooling fan with a large clearance dimension (2.5 cm). Performance improvements were obtained for large flow rates by injection air from fixed shroud to the tip clearance. Niu et al. [22] obtained a reduction in the leakage rate using the effect of injection from different configuration of a row of 10 equidistant holes, oriented 45 deg to the end face of a turbine blades. For shrouded fan, Buisson et al. [23] studied a new casing treatment, which consists on helicoidal grooving of casing. The obtained results showed reduction both of 45% in leakage flow rate and 2.3% in static efficiency, and the increase of 0.4% in torque.

In this study, we investigate experimentally and numerically the effect of the active flow control by air injection in the tip clearance of axial flow cooling fan. This fan is obtained with the rotational molding process, which involve hollow shape for the whole fan (hub, blades and shroud ring). The main aim of this work is to use the hollow shape induced by this process in order to exploit it in the control of tip clearance flow by steady air injection through rotating shroud ring, which is composed of injection holes oriented in such a way to reduce both of leakage flow rate and of the torque. With specific drive system realized at the LIFSE laboratory of Arts et Metiers Institute of Technology and adequate CFD modeling, the obtained results show reduction in leakage flow and gain in the torque.

## 2. Experimental Setup

### 2.1. Blade Geometry

**Figure 1:**
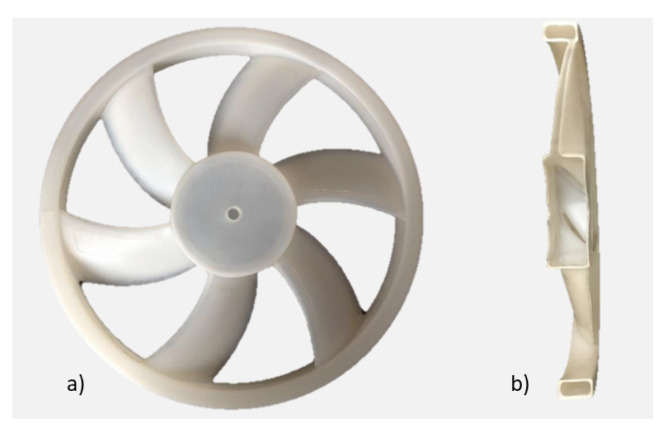
Hollow fan obtained with rotational molding process, (**a**) the whole fan and (**b**) the fan cut in two parts [25].

**Figure 2:**
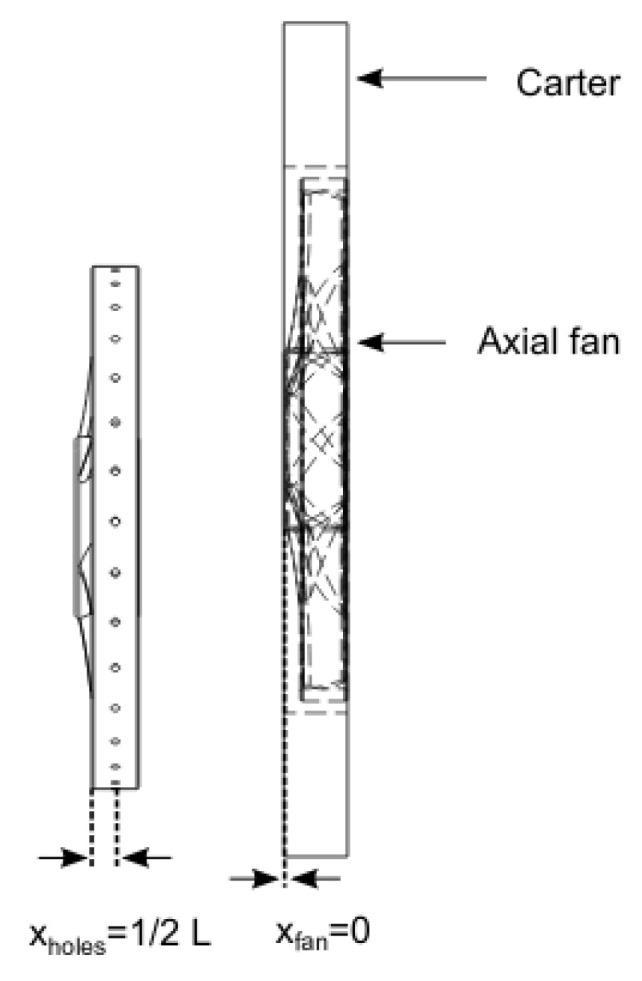
On the left, position of the injection holes on the shroud ring, and on the right, position of the fan in the carter.

In this study, we use an axial-flow cooling hollow fan developed by the rotational molding process using polyethylene material (Figure 1) [24,25], where the preliminary researches have been developed at Arts et Metiers Institute of Technology. This fan was generated following a controlled vortex designed axial-flow [26]. It has six blades, a tip radius $R_{max}=179$ mm and a hub-to-tip radius ratio ($R_{int}/R_{max}$) equal to 0.337 (see Nomenclature). A hollow circular shroud ring of 31 mm length and 9 mm thickness was added to ensure the increase of the fan solidity. The leakage gap has a radial thickness of 4 mm. The 16 injection holes of 4 mm diameter are regularly spaced on the circumference and are oriented in the direction (r,θ,z)=(1,−1,1). The axial direction of the injected air is thus the same as the main discharge flow, and the tangential direction of the injected air is opposed to the fan solid body rotation. Figure 2 shows the location of the injection holes regarding the rotating shroud and the positioning of the latter regarding the casing; the holes are placed in the middle of the shroud and the hub-bowl of the fan is positioned at $x_{fan}=0$. The casing has a width of 46 mm. These positions were investigated and their impact on the fan characteristics are not presented in this paper.

For injection operating condition, we use the injection rate ξ, defined as: $\xi =q_{inj}/q_{max}$, where $q_{inj}$ is the injection rate delivered by the drive system and $q_{max}=1000$ L/min is the maximum injection rate for the fan resistance to the internal flow.

### 2.2. Test Bench and Drive System

**Figure 3:**
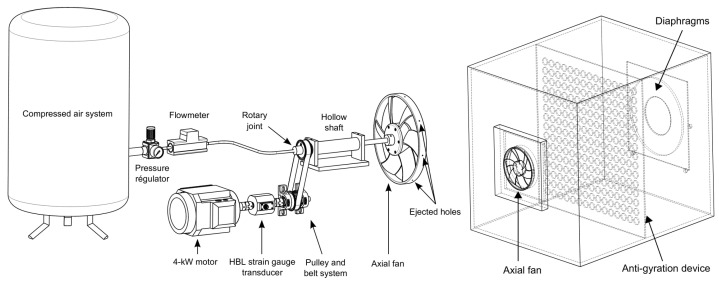
Experimental setup: (**a**) the fan drive system, (**b**) ISO−5801 test bench (dimensions 1.3m×1.3m×1.8 m).

The drive system and the test bench were reported in [24,25]. In brief, Figure 3 presents the test bench used to determine the global performance which is built at the LIFSE laboratory of *Arts et Metiers Institute of Technology* and developed according to the ISO−5801 standard. It consists of a rectangular box in which the pressure rise of the fan is measured. The fan is mounted on one side of the box and is rotated with a shaft, coupled to an electrical motor and a T20W HBM rotating torquemeter. Air is injected into the hollow shaft through a rotary joint Deulin 1115-000-200. The total flow rate accross the fan $q_{v}$ is set and measured according to the ISO−5167 standard with diaphragms of various diameters Φ that are put on the opposite side of the rectangular box. The fan static pressure rise Δp is measured with an absolute precision of 0.1 Pa. For the torque, the uncertainty of the measurements is 0.1% of the full scale equal to 5 N·m.

Initially, in order to investigate the behavior of the fan over a wide range of operating flow conditions, ten diaphragm diameters are tested. The diameters of the diaphragms are reported in Table 1. The measurements taken show that for a diameter Φ⩾238 mm the aeraulic power exhibits notable improvements in proportion to the diameters of the diaphragm.

### 2.3. Torque Measurement

In the present experimental set-up, different components generate an elementary resistive torque. Among these components that are used for the mechanical transmission, one can find for instance the motor shaft, the couplings, the torque meter, the belt and pulleys, the bearings and the rotary joint. It is difficult to estimate separately the contribution of each of these elements. Our goal is to characterize the operation of the fan alone for the different control configurations to get the static efficiency. For a given rotation rate, this amounts to estimating its resistive torque. To do this, we proceeded first to determine two torques, the first one at no load $C_{0}$ (fan removed) and the second one in presence of the fan *C*. The net torque will then be given as follows:

(1) $$C_{net}=C-C_{0}$$

**Figure 4:**
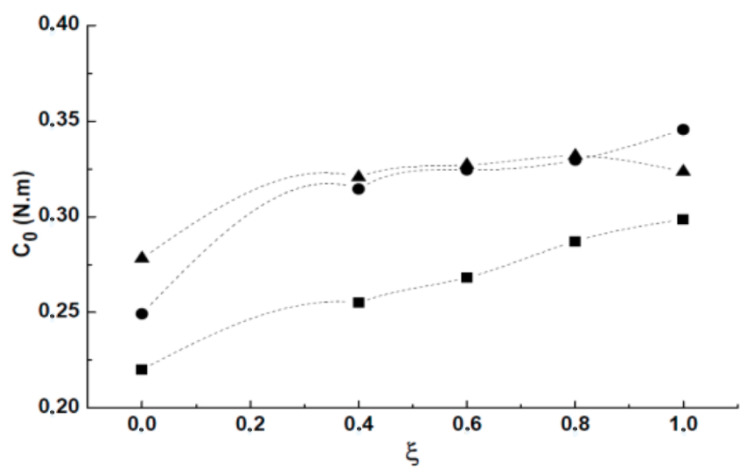
No-load torque variation $C_{0}$: (■) 1000 rpm, (•) 1500 rpm, (▲) 2000 rpm.

During the measurements, we noticed that the no-load torque $C_{0}$ depends on the motor speed and on the injection rate. The Figure 4 shows that for 1500 and 2000 rpm, the torque $C_{0}$ stabilizes from an injection rate of the order of (ξ=0.4). It can be said that there is a saturation of the internal friction generated in the two rotating parts of the mechanical circuit (including the rotary joint and the hollow shaft). However, this phenomenon is not yet achieved for a rotation rate of 1000 rpm.

## 3. CFD Modeling and Grid Generation

The research carried out in numerical modeling has shown that it is difficult to determine all the structures generated in the flow, because it requires time and heavy computational resources. In the field of rotary flows, to simplify the problem, the periodicity condition is often used in order to carry out a detailed study on the area of interest. However, in the present experimental arrangment, periodicity is not applicable due to the number of holes (16) with respect to the number of blades (6) and to the rectangular shape of the test-bench. Thus, the complete geometry of the test bench is taken into account for the simulation.

**Figure 5:**
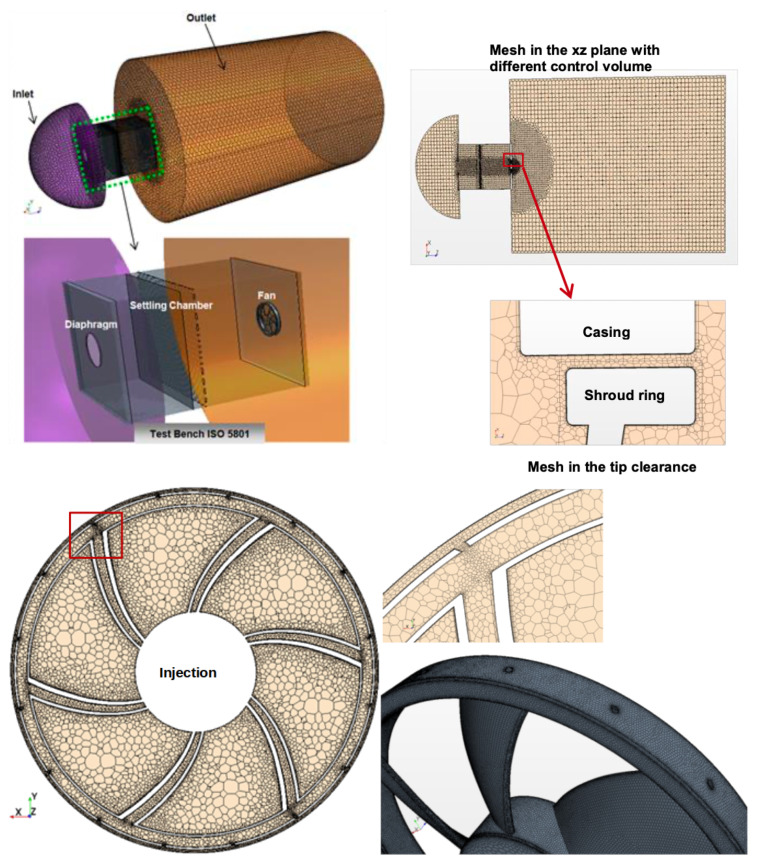
Grid generation for the test bench ISO 5801 and detailed view of the mesh inside the hollow blades.

The generation of structured mesh presents a particular difficulty in the areas close to the settling chamber, the hollow area of the fan and especially at the level of the injection holes. For this, we opted for a polyhedral mesh recognized by these advantages. The refinement criteria were carried out with appropriate control volumes size (boundary layer, wake, pressure gradients, etc.). Figure 5 presents the complete numerical domain generated in polyhedral mesh.

**Figure 6:**
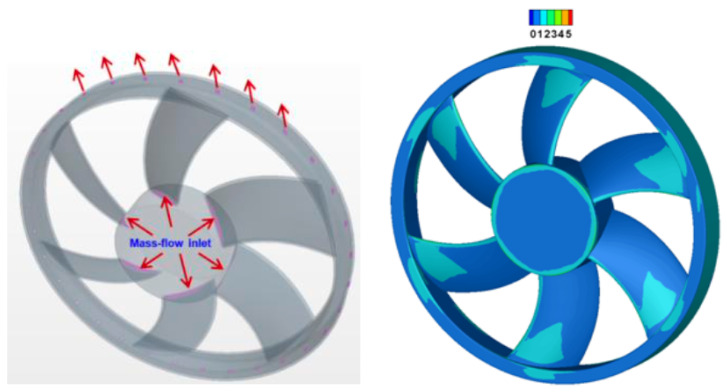
Mass flow rate Boundary condition for air injection in the inside hub and wall $y^{+}$ on the fan.

In our work, we use the commercial code ANSYS-Fluent Fluid Simulation Software, that is based on the finite volume discretization. Our approach is based on the study of the steady viscous incompressible flow with the Menter’s Shear Stress Transport turbulence model (k−ω SST). The Moving Reference Frame (MRF) or Frame motion method is used to model the fan motion. For velocity-pressure coupling, SIMPLE algorithm is used with MUSCL Third order for the momentum equation and the second order for the rest of equations. For boundary conditions, both of inlet and outlet are maintained at atmospheric pressure. For the controlled flow configuration, the air injection is defined by mass flow inlet condition inside the hub as shown in Figure 6. The turbulence intensity has been set to 1% and the turbulent viscosity ratio to 1 on the inlets. A grid convergence test has been performed for the diaphragm of diameter Φ=300 mm and a rotation rate of 1500 rpm. The static pressure rise Δp, the volume flow rate $q_{v}$, the torque *C* and the leakage mass flow rate in the gap are reported in Table 2 for various meshes. The results are considered as converged for grid (4). This is the mesh that is used in the following of the article. The meshing parameters are such that the wall $y^{+}$ on the fan is of the order of 1 for this grid, as can be seen in Figure 6.

## 4. Results

The main findings are presented in term of gain in fan power, torque and leakage flow for the following operation conditions: 16 injection holes, two rotation speed (1000 rpm and 2000 rpm) and two injections rate (ξ=0.4 and ξ=0.8).

### 4.1. Aeraulic Power

We define the power gain as follow: $Gain=\left(P-P_{0}\right)/P_{0}$, where $P=q_{v}\Delta p$ is the delivered power for the controled configuration and $P_{0}$ is the power delivered for $q_{inj}=0$. We discussed the influence of the diaphragm diameter, rotationnal speed and the number of the injection holes (16 and 32) where the active control improves the delivered power nearly up to 40% [24,25]. Other measurements carried out for a series of fans have shown that the optimum gain in fan power varies between 30% and 53%. This wide range of variation results mainly from the operating conditions of the rotational molding and from the manufacturing defects of the fan.

**Figure 7:**
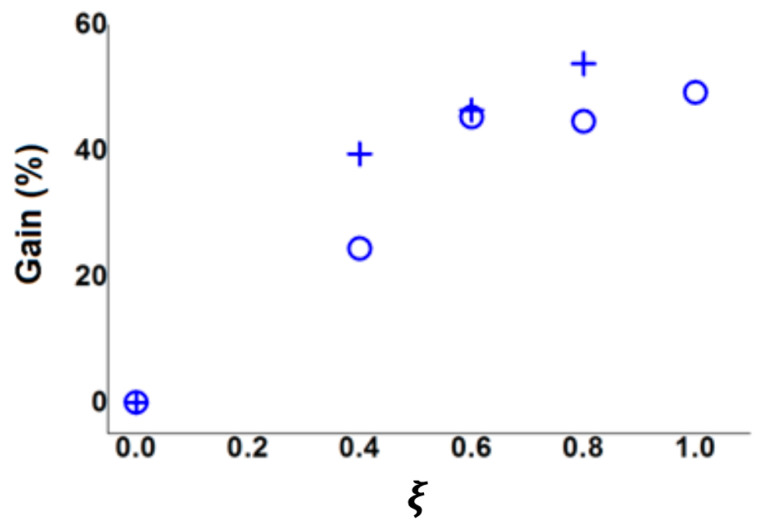
Experimental results of the power gain added by the control for: diaphragm diameter Φ=375 mm, rotation speed 1000 rpm. (+) results for 16 injection holes and (○) results for 32 injection holes.

The Figure 7 shows the maximum gain (53%) obtained for diameter diaphragm of Φ=375 mm, rotation speed of 1000 rpm and 16 injection holes.

### 4.2. Resistant Torque

**Figure 8:**
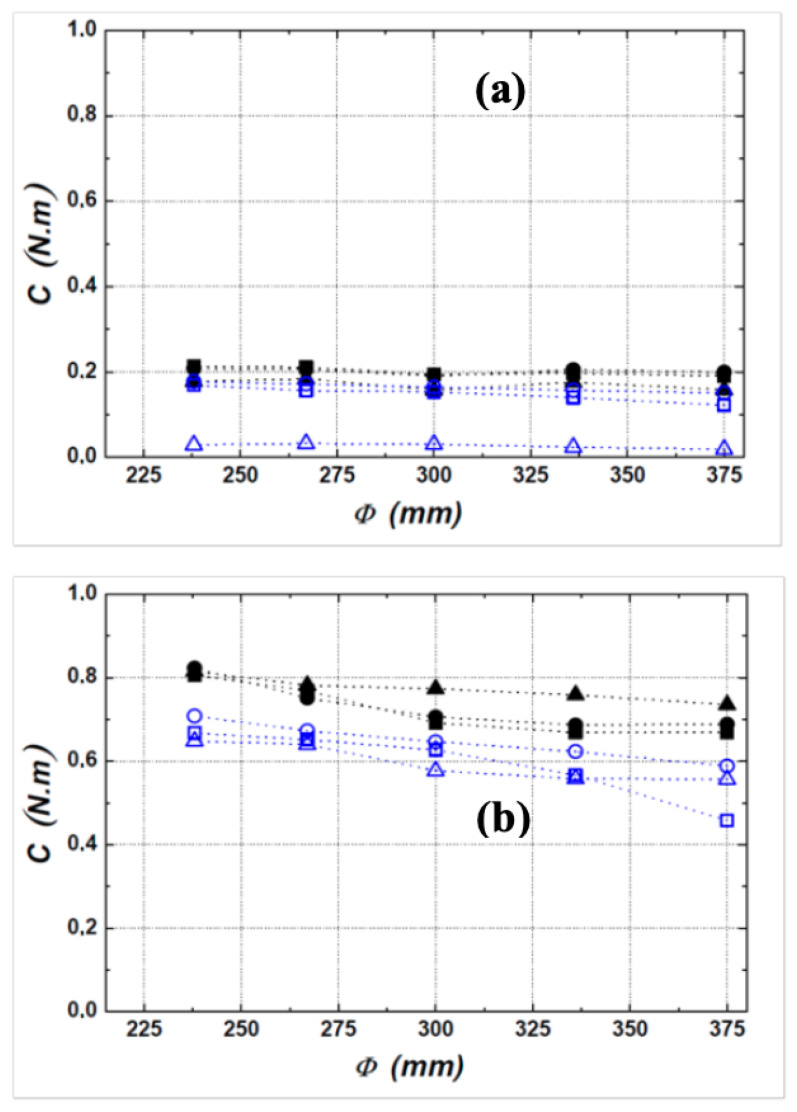
Evolution of the fan torque: (**a**) 1000 rpm and (**b**) 2000 rpm. Experimental results (■) baseline, (•) ξ=0.4, (▲) ξ=0.8, CFD results (☐) with control, (○) ξ=0.4, (△) ξ=0.8.

Figure 8 shows the evolution of the torque developed by the fan for numerical and experimental cases. The values obtained are practically low for the two rotational speeds and seem independent of the diaphragm diameter Φ. However, with a high speed of rotation, these low values have a significant influence on the motive power and therefore the static efficiency.

From the obtained results for the experimental study, it is possible to conclude that for a control by 16 holes a maximum reduction of the torque of about 19% is reached for the speed conditions of 1000 rpm, of the diameter Φ=300 mm and of the injection rate ξ=0.8. The explanation is that the fan is in a rotating sprinkler configuration. The torque becomes important for a high speed of rotation (2000 rpm). In the operating tip clearance, friction remains predominant because the contribution of the blowing in terms of reducing the resistive torque remains minimal.

In the numerical simulation, the maximum torque reductions are estimated at:

- 80% for (1000 rpm, ξ=0.8 and independently of the diaphragm diameter).
- 8% for (2000 rpm, Φ=300 mm and ξ=0.8).

It is noted that the gain in torque obtained experimentally (19%) is relatively low compared to the value of the gain resulting from the simulation (80%). This could be due to several experimental factors including:

1. Sensitivity of the torque meter used (HBM 5 N·m). The measurement range seems important relative to the values that have been measured which are quite small. It is desirable to equip the experimental setup with a more suitable torque meter. In addition, the measurements are not direct due to the size constraint of the assembly. The possibility of positioning the torque meter upstream of the fan (inside the box) has been abandoned due to disturbances induced in the upstream suction flow. In the event that the assembly is carried out downstream of the fan, the torque meter must be equipped with a hollow shaft (to ensure the control of the main flow) with sealed couplings to remedy leaks in the injection flow.
2. Difficulty in determining the elementary torques of assembly components (belt transmission, coupling, rotating joint, etc.). We considered the net torque approach (without and with fan) with the hypothesis that with or without control, the elementary torques remains invariable.

### 4.3. Leakage Flow

**Figure 9:**
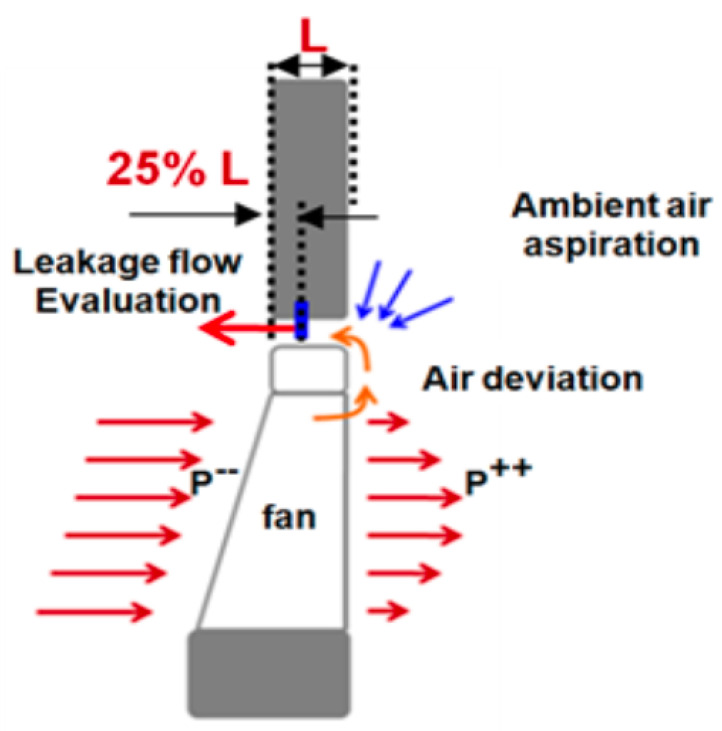
Leakage flow, evaluation and these sources.

The reduction of the leakage flow with evaluation of its influence on the different fan powers constitutes one of the primary objectives of this study [24]. From this perspective, numerical simulation provides access to the evolution of the flow in areas with experimental measurement difficulties. The leakage rate generated in a limited space of 4 mm is a typical illustrative example, where it takes sources from air deviation at the exit blade or the aspiration from the stagnation ambient air. In our work, this flow is calculated at 25% at the exit surface of the leakage flow as shown in Figure 9.

**Figure 10:**
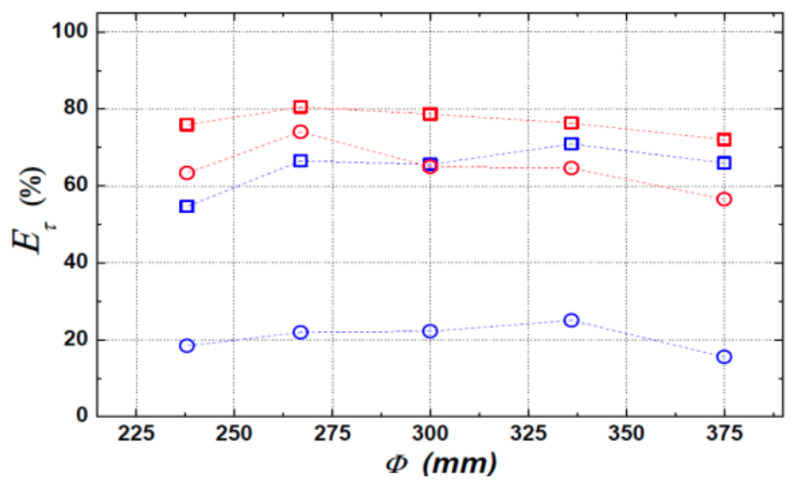
Evolution of the gain in leakage flow by simulation (16 injection holes): 1000 rpm: (☐ in blue color ) ξ=0.4, (☐ in red color) ξ=0.8, 2000 rpm: (○ in blue color ) ξ=0.4, (○ in red color ) ξ=0.8.

In order to estimate the inherent variation of this flow relative to the nominal case, we define the gain in leakage flow as follows:

(2) $$E_{\tau }\left(\%\right)=100\times \frac{{\dot{m}}_{gap_{0}}-{\dot{m}}_{gap}}{{\dot{m}}_{gap_{0}}}$$

where ${\dot{m}}_{gap_{0}}$ is the leakage flow rate calculated for the uncontrolled case and ${\dot{m}}_{gap}$ for the controlled case. The typical order of magnitude of the uncontrolled leakage flow rate is 8% of the air flow rate generated by the fan as can be seen in Table 2. From the Figure 10, the results are presented as follows:

- For the speed of 2000 rpm and an injection rate of ξ=0.4, a specific reduction of about 20% of the leakage flow.
- For the rest of the curves relating to the cases checked, a reduction between 50% and 80% of the leakage rate is achieved. The maximum reduction is obtained for low rotation speed (1000 rpm) and high injection rate ξ=0.8.

**Figure 11:**
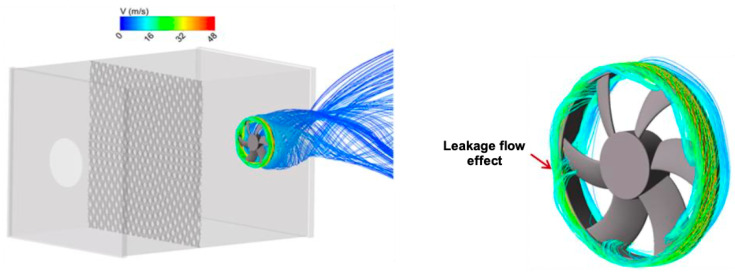
Flow structure near the leakage gap, rotation speed 2000 rpm, diaphragm diameter Φ=375 mm.

**Figure 12:**
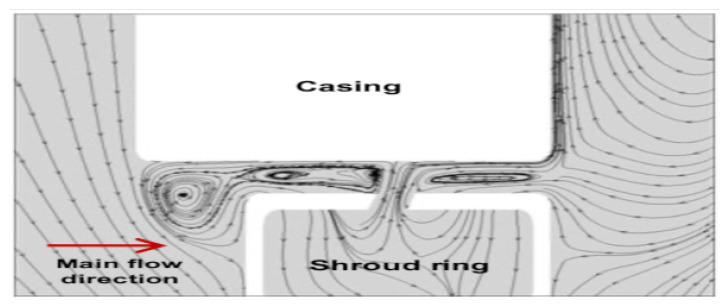
Flow structure for control case near the jet at the meridian plan, rotation speed 2000 rpm, diaphragm diameter Φ=375 mm and ξ=0.8.

It can be seen from Figure 11 characterizing the nominal case, that the leakage flow behavior at tip clearance as well as both of upstream and downstream of the fan, is with high complexity including intense zones of swirling motion. Meanwhile, when the flow control is imparted to the fan (Figure 12) at the flow conditions ξ=0.8, rotation speed 2000 rpm and diaphragm diameter Φ=375 mm, important phenomenon is observed at the tip clearance between the casing and the shroud ring at the injection zone. It consists of two dead counter-rotating air regions leading to formation of two eddies besides the injection stream. Resulting in a blockage of the ambient air aspiration toward the fan portside induced by the pressure drop (Figure 9).

### 4.4. Entropy Analysis

To study the losses production generated by the leakage flow, it is recommended to use the entropy analysis [2,6,27]. It is shown that for turbulent flow with severe complex structure separation, the entropy analysis can be the better method to estimate these losses and to perform the possibilities of selective flow control conditions.

**Figure 13:**
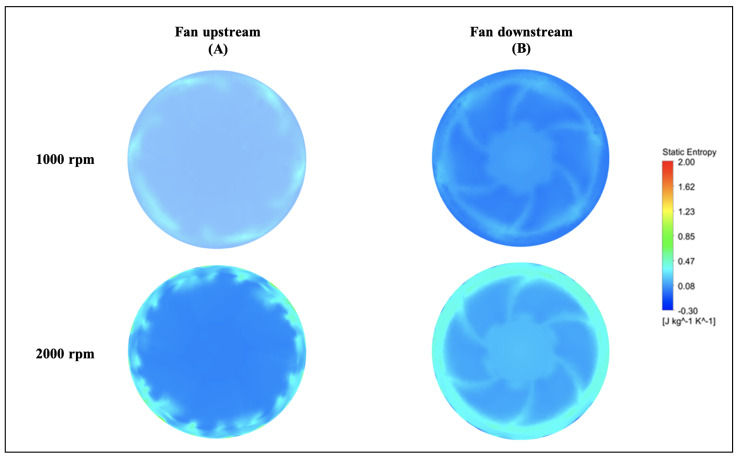
Static entropy distribution for “no control configuration” at fan upstream and downstream for two rotation speeds 1000 and 2000 rpm, for Φ=375 mm.

**Figure 14:**
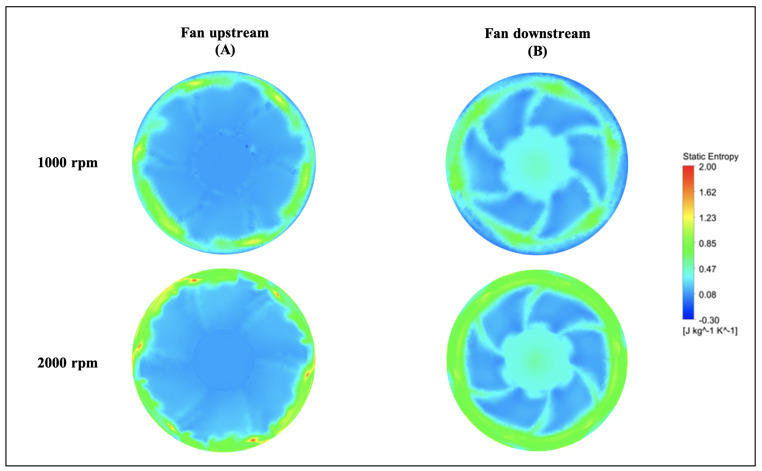
Static entropy distribution for injection rate ξ=0.8 with 16 holes at fan upstream and downstream for two rotation speeds 1000 and 2000 rpm, for Φ=375 mm.

**Figure 15:**
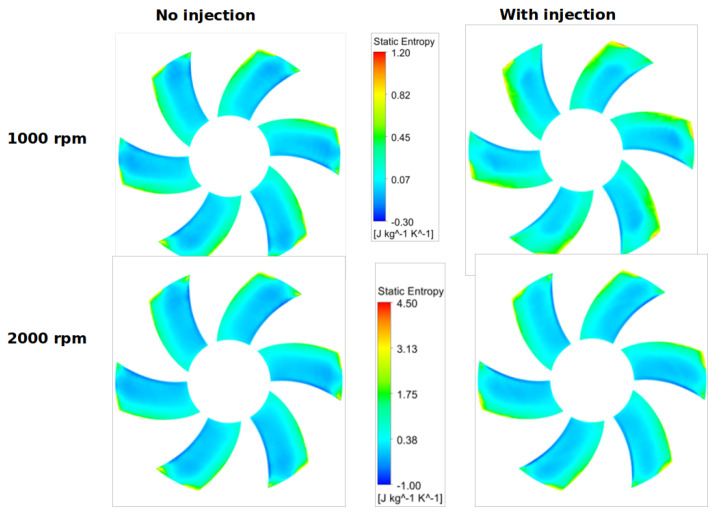
Static entropy distribution on the blades suction side for two rotation speeds 1000 and 2000 rpm, with no injection and ξ=0.8, for Φ=375 mm.

**Figure 16:**
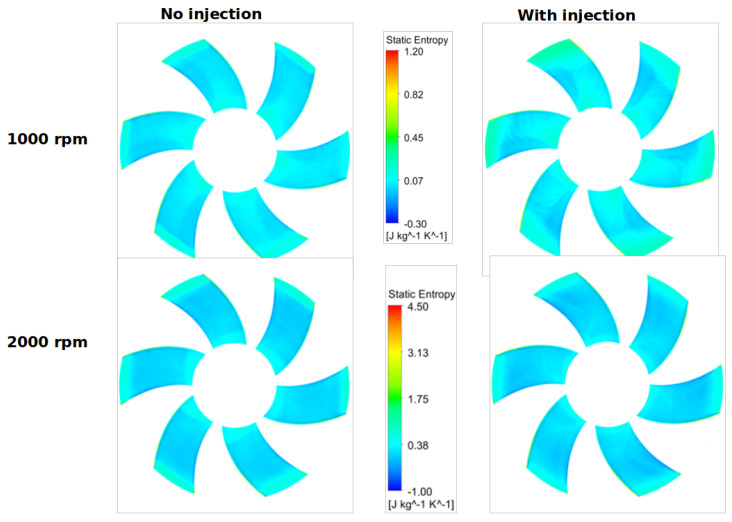
Static entropy distribution on the blades pressure side for two rotation speeds 1000 and 2000 rpm, with no injection and ξ=0.8, for Φ=375 mm.

**Figure 17:**
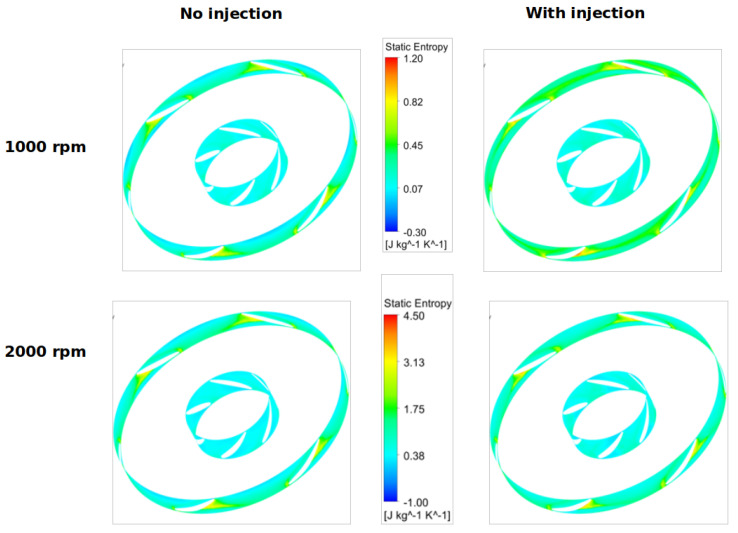
Static entropy distribution close to the hub and shroud for two rotation speeds 1000 and 2000 rpm, with no injection and ξ=0.8, for Φ=375 mm.

For two circular surfaces defined 2 mm upstream and downstream of the fan with radius at the mid-tip clearance, Figure 13 and Figure 14 show the distributions of the static entropy, respectively, for two configurations, no injection and with injection rate ξ=0.8 with 16 holes. The same quantity is displayed on the blades pressure side, suction side and on two surfaces of constant radius in Figure 15, Figure 16 and Figure 17.

As can be seen, and relatively to the lower entropy variation, an increase in entropy is noted at the level of the shroud ring for important rotation speed (2000 rpm) significantly downstream compared to upstream. This is referred to the interaction between the tip clearance flow and the main flow (Figure 11 and Figure 12). For the same reason, a relatively high-level entropy distribution is generated near the shroud ring in injection configuration.

**Figure 18:**
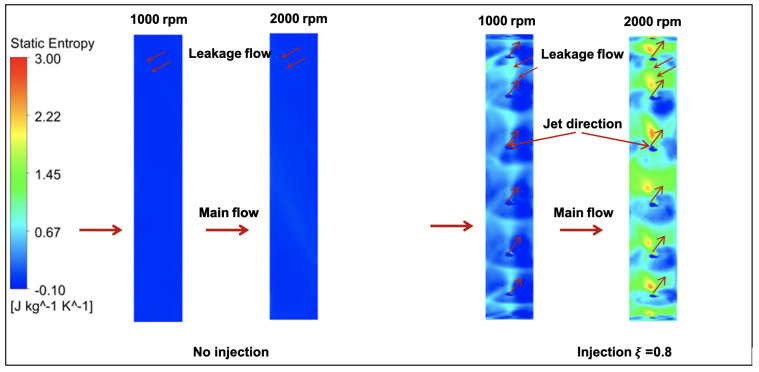
Static entropy distribution at the mid tip clearance with Φ=375 mm.

In the mid tip clearance zone (Figure 18), without control no significant entropy variation is mentioned. However, with control flow configuration and near the injection holes, a circumferential alternative increase and decrease of the entropy is obtained. This is due to the separation phenomena induced by the local jet which can be analyzed by similarity to the structure of the Jet In Cross Flow (JICF). The increase of the entropy is located in the bending direction of the jet where the momentum injection is important. The structure of the (JICF) is characterized by a complex three-dimensional vortex system especially in the tip clearance and shows in practical its ability to mix the leakage flow and to introduced, if we can say, a controlled jet force.

## 5. Conclusions

An axial flow fan was built with rotational molding process. This fan presents hollow blades and a hollow peripheral rotating shroud. This geometic property is used to inject air into the tip clearance through the hub of the fan. The aim of this air injection is to control the leakage rate, a source of energy dissipation. The injected air flow, generated by a specific drive system, exits at the fan periphery by 16 holes, disposed on the shroud. Numerical and experimental results are presented for two rotational speeds 1000 rpm and 2000 rpm and two injection rates $q_{inj}=400$ L/min and 800 L/min.

Experimentally, a gain in the aerodynamic power that the fan delivers is noticied with the increase in the injection rate. At 1000 rpm, for high flow rate of fan, and for an injection rate of 800 L/min the observed gain is 53%. Gains of the same order of magnitude, i.e., from 30% to 50% are also observed at higher rotation rates and lower injection rates [25]. This technology thus seems promising to enhance the performances of axial-flow fans that are used in in the heating, ventilation and air conditioning industry or in automotive engine cooling.

The numerical simulations that are presented in this article give new insight into the phenomenon. The computational fluid dynamics tool allows access to the velocity fields in the gap between the rotating shroud and the carter, which could not be possible in a reasonably easy way by experimental means. The simulations confirm the gain in aerodynamic power. The total flow rate that passes through the fan system can be decomposed into two contributions: the main flow to which the fan blades supply energy, and the leakage flow that recirculates in the clearance gap. This last flow is opposed to the main flow and is a source of energy degradation. The results of the numerical study show a significant reduction in the leakage rates for all the operating points of the fan, in a quantitative way. For the two injection rates that have been tested, the reduction in leakage flow rate is of the order of 60% and 80% for the rotation rate of 1000 rpm, and of the order of 20% and 60% for the rotation rate of 2000 rpm. Moreover, a small gain in power consumption is also reported, but the numerical simulations indicate that the reduction of the leakage flow rate is the main source of the aerodynamic power enhancement.

## Figures and Tables

**Table 1:** Diaphragm’s diameters tested.

Φ **(mm)**	77	151	169	190	220	238	267	300	336	375

**Table 2:** Grid convergence test, for Φ=300 mm and 1500 rpm, with no control.

Grid	Rotating Domain	Fixed Domain	Δp	$q_{v}$	*C*	${\dot{m}}_{\mathrm{gap}0}$
	($\times 10^{6}$ cells)	($\times 10^{6}$ cells)	(Pa)	(m${}^{3}$·s${}^{-1}$)	(N·m)	(kg·s${}^{-1}$)
(1)	0.130	0.731	64	0.459	0.399	0.0378
(2)	0.532	1.678	52	0.408	0.345	0.0378
(3)	1.548	3.462	55	0.412	0.357	0.0414
(4)	2.227	4.935	55	0.411	0.352	0.0408
(5)	8.205	6.298	55	0.409	0.353	0.0402

## Data Availability

The data presented in this study are available on request from the corresponding author.

## Nomenclature

The following abbreviations are used in this manuscript:

$C_{0}$	Torque without control
*C*	Torque with control
$C_{net}$	Net torque
Gain	Power gain added by the control
$q_{inj}$	Injection rate m${}^{3}$·s${}^{-1}$
$q_{max}$	Maximum injection flow rate m${}^{3}$·s${}^{-1}$
$q_{v}$	Fan flow rate m${}^{3}$·s${}^{-1}$
*P*	Power delivered by the fan [*W*]
$P_{0}$	Power delivered by the fan for $q_{inj}=0$
$R_{int}$	Hub radius [m]
$R_{max}$	Tip radius [m]
${\dot{m}}_{gap_{0}}$	leakage flow gap rate without control
${\dot{m}}_{gap}$	leakage flow gap rate with control
Δp	Pressure difference generated by the fan [Pa]
ξ	Injection rate
Φ	Diaphragm diameter [m]
$x_{fan}$	Relative position between the fan and the carter [m]
$x_{holes}$	Position of the injection holes on the casing [m]
